# Multimodal assessment of liver stiffness and portal hemodynamics in a cirrhotic mouse model

**DOI:** 10.3389/fbioe.2026.1888436

**Published:** 2026-08-03

**Authors:** Jingtian Zhang, Yujie Huo, Jingxuan Bian, Li Cha, Qiyue Yang, Chunjuan Zhao, Jinyu Li, Dongliang Zhao, Duiping Feng, Long Gao

**Affiliations:** 1 Department of Oncological and Vascular Intervention, First Hospital of Shanxi Medical University, Taiyuan, Shanxi, China; 2 Department of Medical Imaging, Shanxi Medical University, Taiyuan, Shanxi, China; 3 Institute of Biomedical Engineering, Shenzhen Bay Laboratory, Shenzhen, Guangdong, China

**Keywords:** hepatic fibrosis, mechanobiology, non-invasive diagnosis, portal hemodynamics, second harmonic generation

## Abstract

**Background and aims:**

Elevated pressure within the portal venous system defines portal hypertension. While extensive research has focused on its biochemical basis, the biomechanical characteristics of liver tissue in this condition remain largely unexplored. We established a carbon tetrachloride (CCl_4_) -induced murine model to investigate dynamic changes in liver biomechanics and their associations with hemodynamics, fibrosis, and hepatocellular injury.

**Methods:**

Portal hypertension was induced in six-week-old male BALB/c mice by intraperitoneal CCl_4_. Ultrasound measured surrogate hemodynamic indices such as resistance index, flow velocity, and spleen size. Uniaxial tensile testing obtained liver Young’s modulus. Second harmonic generation and histology evaluated collagen microstructure. Serum biomarkers by enzyme-linked immunosorbent assay.

**Results:**

Collagen deposition was significantly and positively correlated with liver stiffness (r = 0.7232; p < 0.0001). Liver stiffness also correlated with the portal venous pulsatility index (r = 0.6477, p = 0.0005). Serum biomarkers, including aspartate aminotransferase (AST) and alanine aminotransferase (ALT), were positively correlated with liver stiffness (AST: r = 0.6596, p = 0.0016; ALT: r = 0.8451, p < 0.0001). Expression levels of α-smooth muscle actin (α-SMA) and Matrix Metallopeptidase-9 (MMP-9) were significantly associated with increased stiffness (α-SMA: r = 0.6837; p = 0.0002; MMP-9: r = 0.7365; p < 0.0001).

**Conclusion:**

In CCl_4_-induced cirrhosis, liver stiffness correlates temporally with portal resistance, collagen deposition, and HSC activation, suggesting a potential mechanobiological contribution to portal hypertension pending causal validation, with interrelated hepatocellular injury, inflammation, and ECM deposition.

## 1 Introduction

Portal hypertension is common in cirrhosis (de Franchis et al., 2022) and is traditionally attributed to vasoactive imbalances that increase intrahepatic resistance and splanchnic circulation (Li et al., 2025). Recent studies suggest that pathological collagen deposition and cross-linking contribute to the progression of portal hypertension (Gan et al., 2024). Specifically, during portal hypertension, liver tissue stiffness, quantified using Young’s modulus, increases. This mechanical change likely contributes to elevated pressure in the portal vein (Mazumder et al., 2023). Increased liver stiffness may be a critical mechanical determinant of pathological changes in the portal system (Long et al., 2025). However, current clinical and research paradigms mainly regard liver stiffness as a consequence of fibrosis (Reiter et al., 2021), while its role as a driver of portal hypertension progression remains unexplored. Furthermore, it is unclear whether macroscopic tissue mechanics are directly associated with hemodynamics and microscopic cellular mechanical signals and which mechanisms mediate this relationship. Macroscopic mechanical changes are likely mediated by abnormal remodeling of the extracellular matrix (ECM)(Naba, 2024; Ahn and Park, 2026) and can promote sustained activation of hepatic stellate cells through mechanosensitive signaling pathways, forming a feedback loop between mechanical forces and pathology (Felli et al., 2023; Zhang et al., 2024).

To address these questions, we established a mouse model of portal hypertension and used a multiscale multimodal approach to integrate *in vivo* ultrasound hemodynamic assessment, *ex vivo* liver tensile mechanics testing, second harmonic generation (SHG) imaging, and molecular analyses. This study examined how macroscopic liver mechanics relate to portal hemodynamics, extracellular matrix remodeling, and cellular mechanotransduction, offering empirical support for a mechanobiological paradigm in portal hypertension and guiding therapies that target mechanotransduction pathways.

## 2 Materials and methods

### 2.1 Animal experiments

A total of 35 male BALB/c mice (6 weeks old) were obtained from Guangdong Weitong Lihua Laboratory Animal Technology and housed at the Shenzhen Bay Laboratory Animal Research Center (the facility where all animal husbandry and experimental procedures were conducted). The animals were kept under standard housing conditions (12 h light/dark cycle) with free access to water and a regular rodent chow diet. All experimental procedures were carried out in line with institutional guidelines and received approval from the Laboratory Animal Welfare and Ethics Committee of Shenzhen Bay Laboratory (approval No. AEZDL202501). Mice were allocated to experimental groups using a randomization procedure, and outcome assessments were performed by investigators blinded to group assignment.

To induce liver cirrhosis and portal hypertension, the experimental animals received intraperitoneal injections of a CCl_4_-olive oil mixture (4: 1 (v/v)). The initial dose of the CCl_4_ mixture was 5 mL/kg, followed by injections every other day at 2.5 mL/kg. Control mice received an equal volume of olive oil alone. Seven experimental groups were formed by random assignment of the mice (n = 5 per group) before the first injection: Control (untreated, week 0), Control-2W (olive oil only, week 2), Control-4W (olive oil only, week 4), CCl_4_-1W, CCl_4_-2W, CCl_4_-3W, and CCl_4_-4W. The two olive oil control groups were used to assess potential time-dependent effects of the vehicle.

### 2.2 Sample collection

At specified time points, mice underwent ultrasound imaging, followed by weighing. Deep anesthesia was then induced in mice by intraperitoneal injection of tribromoethanol (250 mg/kg). After loss of reflexes, blood was collected via cardiac puncture. Subsequently, liver and spleen tissues were harvested, and wet weights were recorded. The left lateral lobe of the liver was trimmed into uniformly sized tissue blocks: a portion was used for uniaxial tensile testing and another portion was fixed in 4% paraformaldehyde for staining and SHG imaging.

### 2.3 Abdominal ultrasound

Serial abdominal ultrasound examinations were carried out at weeks 1, 2, 3, and 4 using a dedicated small-animal ultrasound system (Vevo 3100, FUJIFILM VisualSonics) fitted with a 40–55 MHz linear probe (MS550D MicroScan). The mice were anesthetized with 1% isoflurane and 99% oxygen. The mice were positioned supine on a temperature-controlled heating pad, and anesthesia was maintained using 0.5%–1% isoflurane. After depilation, ultrasound gel was applied, and the probe was placed subxiphoid to visualize the portal vein in pulsed Doppler mode. Spleen size was measured transversely, recording the maximum vertical distance from the anterior to the posterior capsule, perpendicular to the long axis. We rely on portal venous pulsatility index (PVPI) (Baikpour et al., 2020; Lee et al., 2024) and changes in diameter and flow velocity, which are highly correlated with portal vein pressure in rodents with cirrhosis (Königshofer et al., 2018; Chen et al., 2022). The measurement is done in the portal vein, using a fixed angle of incidence (≤60°) and averaging over three consecutive cardiac cycles, and the average values were calculated for analysis.

After obtaining a clear and stable pulsed-wave Doppler spectrum of the portal vein, the resistance index was automatically calculated by the built-in software of the ultrasound system. The portal venous pulsatility index (PVPI) was calculated using the following formula:

$$\text{PVPI}=\frac{\left(V_{\text{max}}-V_{\text{min}}\right)}{V_{\text{max}}}$$

where V_max_ is the maximum velocity and V_min_ is the minimum velocity of the portal venous waveform over a cardiac cycle.

### 2.4 Uniaxial tensile testing

Liver specimens were obtained from the left lateral lobe and then trimmed. The actual dimensions of each sample were recorded and averaged. Across all specimens, the mean (±SD) values were 6.1 ± 0.18 mm (length), 5.89 ± 0.30 mm (width), and 2.03 ± 0.08 mm (thickness), corresponding to coefficients of variation of approximately 3.0%, 5.1%, and 3.9%, respectively. The specimens were mounted in a multiscale micro-multi-axis mechanical testing system (CARE Measurement & Control Co. Ltd.; IPBF-30S). The specimens were secured using hook-shaped clamps that engaged the tissue ends without crushing the central gauge region. Slip events were monitored in real time through the CARE software interface by closely observing the force-displacement or stress-strain curves throughout each test. The appearance of sudden, discontinuous stress drops (serrated fluctuations) or pronounced changes in slope on an otherwise smooth curve was taken as the primary indicator that microscopic plastic deformation events, such as slip, had occurred within the material; such traces were excluded from the final modulus calculation. The stretching axis is always aligned with the longitudinal axis of the left leaf to minimize anisotropy. The samples were then submerged in PBS to maintain moisture content. Pre-loading was performed at 0.001 mm/s to a load of 0.001 N to eliminate tissue relaxation. The formal tensile test was conducted at 0.01 mm/s up to a 12% strain level. The displacement (ΔL) and load (F) were continuously recorded during the test. The strain rate was approximately 0.00167 s^-1^, calculated from the stretching speed (0.01 mm/s) and the initial sample length (L_0_ ≈ 8 mm). The cross-sectional area was calculated as the product of the average width and thickness. To ensure data reliability, the instrument was calibrated before each batch of tests.

Biological soft tissues typically exhibit a toe region (0%–5% strain) during the initial stage of tensile testing (Ji et al., 2022), reflecting gradual straightening of collagen fibers. The 5%–10% strain interval was verified to be linear for all specimens (R^2^ > 0.95) (Kemper et al., 2010). See the Supplementary Figure S2 for details. Therefore, Young’s modulus was determined as the slope of this linear region. This approach is consistent with previous biomechanical studies that use a linear elastic approximation within defined small-strain windows for comparative purposes. Based on the linear characteristics of the stress-strain curves in this study and following previous literature (Estermann et al., 2021) we selected the linear elastic region (5%–10% strain) to calculate Young’s modulus (E, kPa) from the slope of the stress-strain curve. All samples from each group were tested on the same day.

The cross-sectional area of the sample prior to tensile testing was calculated using the average width and average thickness of the sample:

$$A=\bar{y_{0}}\times \bar{d_{0}}$$

Strain ε reflects the degree of tissue deformation and is calculated as ΔL divided by the initial length (L_0_):

$$\varepsilon =\frac{\Delta L}{L_{0}}$$

Stress φ was calculated as F divided by the initial cross-sectional area of the sample:

$$\varphi =\frac{F}{A}$$

Specimens were consistently trimmed along the same anatomical axis (left lateral lobe, longitudinal axis) for all samples to minimize variability in fiber orientation relative to the loading direction.

### 2.5 Histopathological evaluation

After fixation in 4% paraformaldehyde and paraffin embedding, liver and spleen were sectioned (4 µm and 2 μm, respectively) and stained with hematoxylin and eosin (H&E) for routine morphological assessment.

For collagen deposition analysis, Masson’s trichrome staining was performed using a commercial kit following the manufacturer’s instructions. The stained sections were examined under a light microscope, and images were captured for analysis.

### 2.6 Immunohistochemical staining

Refer to the Supplementary Materials for a comprehensive description of sample handling and image acquisition.

### 2.7 Second harmonic generation (SHG) imaging

Paraffin-embedded liver sections were baked at 60 °C, deparaffinized and embedded in neutral resin. Unstained sections were imaged using a second-harmonic generation (SHG) microscope (FVMPE-RS; Olympus, Tokyo, Japan) to observe collagen. The laser excitation wavelength was set to 900 nm, with a detection wavelength range of 410–460 nm. The acquisition parameters were 1024 × 1024 pixels, with a Z-axis interval of 2 µm per image, resulting in a total imaging depth of 15 µm. For the semi-quantitative analysis of the SHG images, 3 random fields per section (each 1024 × 1024 pixels) were captured. Vascular regions were defined as areas containing portal tracts or central veins with visible luminal structures; avascular regions were selected from parenchymal areas without visible vessels. Both types of regions were selected from the same mouse section to minimize inter-section variability.

### 2.8 Image quantification

Threshold segmentation was performed using ImageJ (version 1.54p, for Windows 64-bit, National Institutes of Health, USA) to calculate the percentage of positive pixels relative to the total number. Please see the Supplementary Materials section for details.

### 2.9 Serum analysis

At the time of tissue collection, blood samples were obtained and then centrifuged at 4,500 rpm for 15 min at 4 °C (Super H16R centrifuge). The resulting serum was aliquoted and kept at −80 °C until further processing. A fully automated veterinary chemistry analyzer (BS-240VET; Mindray Bio-Medical Electronics Co., Ltd.) was employed to measure routine biochemical markers, namely alanine aminotransferase (ALT), aspartate aminotransferase (AST), total bilirubin (TBIL), and direct bilirubin (DBIL). Concentrations of type III procollagen (PC-III), hyaluronic acid (HA), ammonia, interleukin-1β (IL-1β), interleukin-6 (IL-6), tumor necrosis factor-α (TNF-α), transforming growth factor-β (TGF-β1), and thrombospondin-1 (TSP-1) in serum were determined using commercial enzyme-linked immunosorbent assay (ELISA) kits, following the manufacturers’ protocols. Additional information regarding the specific kits used is provided in the Supplementary Materials.

Among the 35 mice initially included (n = 5 per group), samples were excluded from serum analysis due to visible hemolysis, resulting in a final sample size of n = 4 in some groups. Exclusion decisions were made at the sample-processing stage immediately after collection and prior to any downstream processing, and were independent of group allocation and all experimental outcomes. All assessments were performed blinded to group identity.

### 2.10 Statistical analyses

All statistical computations were carried out with GraphPad Prism (version 10.1.2; GraphPad Software, CA, USA). For comparisons involving more than two groups, we performed a one-way analysis of variance (ANOVA) and then applied Tukey’s *post hoc* test for pairwise intergroup comparisons. Normality of continuous variables was assessed using the Shapiro-Wilk test. The test results (W and p values) for all variables involved in correlation analyses are provided in Supplementary Table S1. Variables that met the normality assumption (p > 0.05) were analyzed using Pearson’s correlation coefficient; otherwise, Spearman’s rank correlation coefficient was reported. A two-tailed p-value below 0.05 was regarded as statistically significant for all analyses.

To account for multiple comparisons across the 16 correlations tested, we applied the Benjamini-Hochberg false discovery rate (FDR) procedure with a cutoff of q < 0.05. All statistical analyses were performed using R version 4.5.2 (R Foundation for Statistical Computing, Vienna, Austria), with the p. adjust function. For each correlation, the FDR-adjusted q-value was calculated; associations with q < 0.05 were considered statistically significant after correction. All correlations that were significant at p<0.05 remained significant after correction, with adjusted q -values ranging from 0.00032 to 0.0455.

## 3 Results

### 3.1 Model validation and mechanical phenotypes

#### 3.1.1 CCl_4_-induced liver fibrosis and portal hypertension model: organ indices and histopathology

**FIGURE 1:**
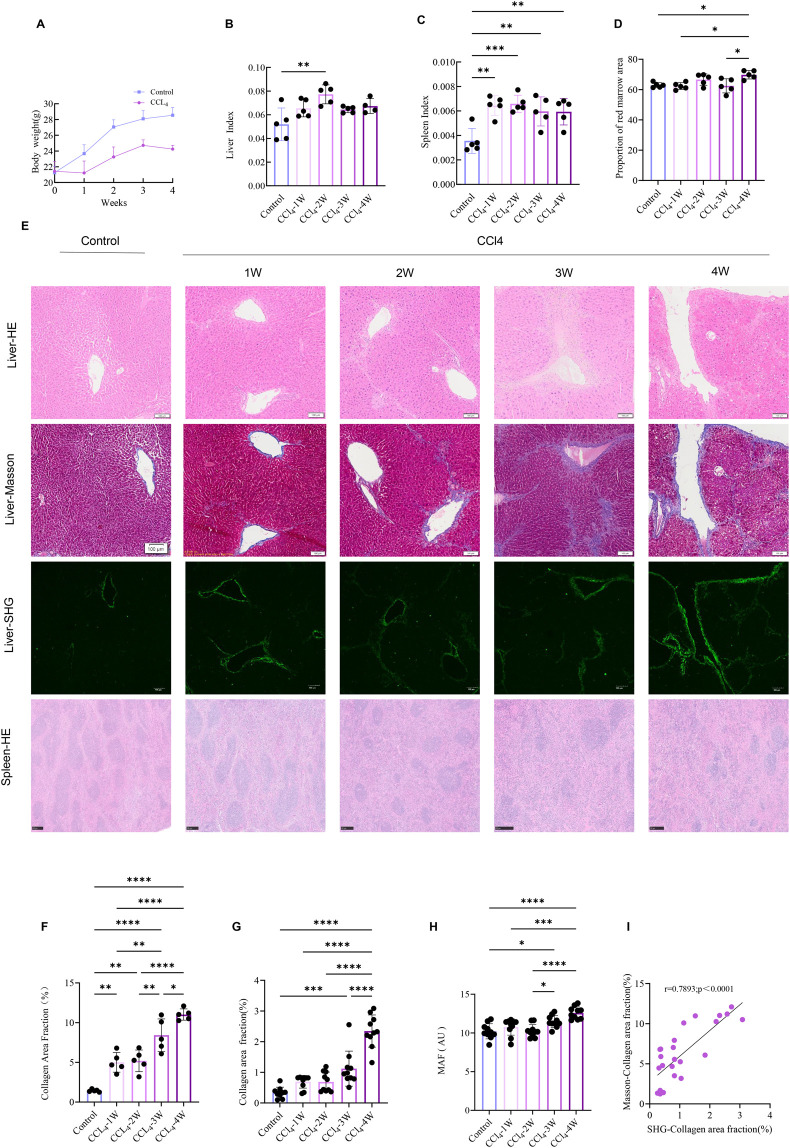
Changes in body weight, staining patterns, and quantification of positive areas during CCl_4_ treatment. **(A)** Body weight. **(B)** Liver index, calculated as (liver weight/body weight) × 100%. **(C)** Spleen index, defined as (spleen weight/body weight) × 100%. **(D)** Quantitative analysis of red pulp area proportion in the spleen using HE staining. **(E)** Representative images of liver histopathological changes (HE, Masson’s trichrome, SHG) and spleen histopathology (HE) at different time points. **(F)** Quantitative analysis of collagen area proportion in the liver using Masson’s trichrome staining. **(G)** Semi-quantitative analysis of collagen area fraction by SHG imaging in vascular and avascular regions of the same mouse liver. **(H)** Semi-quantitative analysis of MAF in vascular and avascular regions of the same mouse liver. **(I)** Correlation analysis between SHG and Masson’s collagen area proportions. Values are expressed as mean ± standard deviation (SD), with n = 5 per experimental group. Intergroup comparisons were performed by one-way ANOVA, and Tukey’s *post hoc* test was applied for pairwise differences. All reported correlations remained significant after Benjamini–Hochberg false discovery rate correction ( q<0.05 ). For correlation analysis, variables with a normal distribution were evaluated using Pearson’s correlation coefficient, while non-normally distributed variables were analyzed using Spearman’s rank correlation coefficient. Significance levels are indicated as follows: ^∗^p < 0.05; ^∗∗^p < 0.01; ^∗∗∗^p < 0.001; ^∗∗∗∗^p < 0.0001. CCl_4_, carbon tetrachloride; HE, hematoxylin and eosin; Masson, Masson’s trichrome staining; SHG, second harmonic generation; MAF, mean autofluorescence intensity.

During CCl_4_ induction, the body weight gain of CCl_4_-treated mice was slowed and then decreased compared to the control group (Figure 1A). The liver index peaked at week 2 before declining (Figure 1B), whereas the spleen index remained elevated throughout the study (Figure 1C). No significant differences in liver and spleen indices were detected among the olive oil-treated control mice at weeks 0, 2, and 4 (Supplementary Figures S1A, B).

#### 3.1.2 Histopathological staging of fibrosis and changes in collagen distribution

To confirm the efficacy of the CCl_4_-induced portal hypertension model, we conducted a comprehensive histopathological assessment incorporating HE and Masson’s trichrome staining and quantified the percentage of positive area.

To assess the extent of splenic congestion, spleen samples from all experimental groups were embedded in paraffin, sectioned, and then stained with HE. The ratio of the red pulp area increased with the duration of modeling (Figure 1D). At week 3, the red pulp area ratio was significantly lower than that observed at week 4. (p = 0.0139), week 1 (p = 0.0112), and in the control group (p = 0.0228) (Figure 1D). No significant differences in the red pulp area of spleen sections were detected among the control groups (Supplementary Figures S1C, D).

Using HE staining as the definitive standard, we documented the pathological progression from inflammatory necrosis to the formation of pseudolobules. HE staining of the liver (Figure 1E) demonstrated that in control mice, the hepatic lobule architecture remained intact, and hepatocytes were radially arranged around the central vein. In contrast, CCl_4_-treated mice exhibited inflammatory necrosis in normal liver tissue, with the lobular structure gradually disintegrating and being replaced by pseudolobules. Additionally, varying degrees of ballooning, steatosis, and focal necrosis were observed (Figure 1E). Liver sections stained with HE from olive oil-treated mice at weeks 0, 2, and 4 displayed preserved hepatic lobular architecture, with no evidence of hepatocellular necrosis, inflammatory infiltration, or fibrosis (Supplementary Figure S1C).

The semi-quantitative statistical results of Masson’s trichrome staining indicated a significant time-dependent increase in the collagen area fraction (Figure 1F). No significant differences in collagen area fraction were observed among weeks 0, 2, and 4 (Supplementary Figure S1E).

#### 3.1.3 Dynamic evolution of collagen microstructure based on SHG

To quantitatively evaluate collagen deposition, we performed SHG imaging on liver sections to measure the collagen area fraction. In addition, two-photon excited autofluorescence imaging was used to assess the mean autofluorescence intensity, which served as a reference for tissue autofluorescence background.

Semi-quantitative analysis indicated a significant increase in the collagen area fraction at week 4 following carbon tetrachloride (CCl_4_) induction compared to the other groups (Figure 1G). Over the experimental period, the mean autofluorescence intensity in the livers of CCl_4_-treated mice rose significantly (Figure 1H). Semi-quantitative analysis of Masson staining corroborated the SHG findings (r = 0.7893, p < 0.0001) (Figure 1I), thereby validating the reliability of SHG-based collagen quantification. SHG imaging of liver sections from olive oil-treated mice revealed sparse, thin collagen fibers, with no evidence of bridging structures or septa formation at any examined time point. Quantitative analysis of the SHG-positive area fraction and mean autofluorescence intensity demonstrated no statistically significant differences among weeks 0, 2, and 4 (Supplementary Figures S1F, G).

### 3.2 Increased young’s modulus is associated with increased hepatic collagen area

**FIGURE 2:**
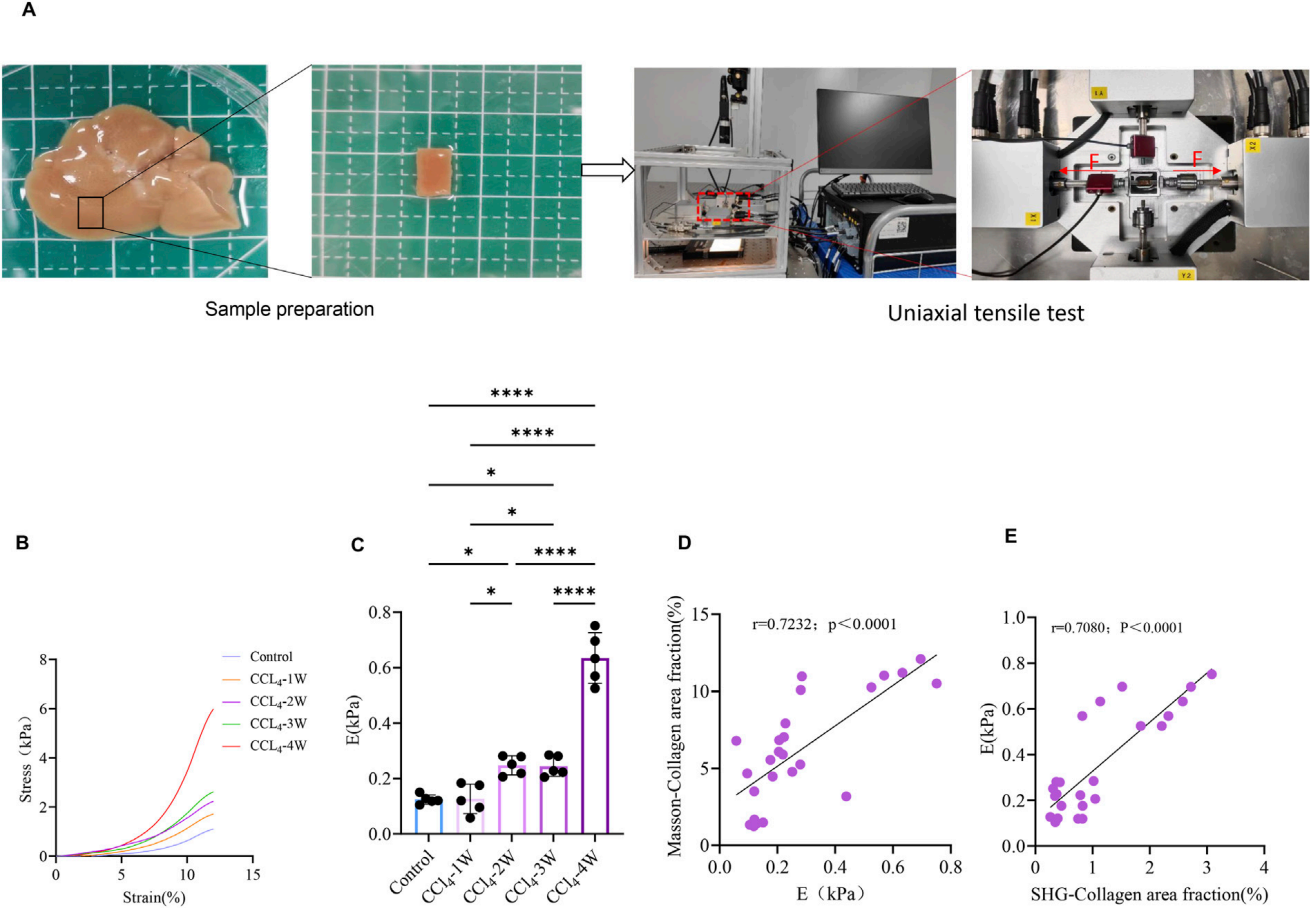
Process and results of uniaxial tensile testing of liver tissue. **(A)** Preparation of liver tissue samples and stretching process. In the figure, F and the arrows indicate the stretching direction. **(B)** Stress-strain curve. **(C)** Statistical results of Young’s modulus. **(D)** Correlation between Young’s modulus and collagen area proportion as determined by Masson’s staining. **(E)** Correlation between the fraction of SHG-positive collagen area and Young’s modulus. Data shown as mean ± SD (n = 5 per group). One-way ANOVA with Tukey’s *post hoc* test was used for group comparisons. For correlation analysis, variables with a normal distribution were evaluated using Pearson’s correlation coefficient, while non-normally distributed variables were analyzed using Spearman’s rank correlation coefficient. ^∗^p < 0.05; ^∗∗^p < 0.01; ^∗∗∗^p < 0.001; ^∗∗∗∗^p < 0.0001. E, Young’s modulus.

To quantitatively assess the biomechanical properties of hepatic tissues, we conducted uniaxial tensile testing on liver samples (Figure 2A). As the disease progressed, the stress-strain curve exhibited an upward and leftward shift (Figure 2B), with a gradual increase in Young’s modulus(E), which reached a significantly higher level than that of all other groups. (Control vs CCl_4_-2W: p = 0.0115; Control vs CCl_4_-3W: p = 0.0141; Control vs CCl_4_-4W: p < 0.0001; CCl_4_-1W vs CCl_4_-2W: p = 0.0133; CCl_4_-1W vs CCl_4_-3W: p = 0.0163; CCl_4_-1W vs CCl_4_-4W: p < 0.0001; CCl_4_-2W vs CCl_4_-4W: p < 0.0001; CCl_4_-3W vs CCl_4_-4W: p < 0.0001) (Figure 2C). To eliminate potential time-dependent effects of olive oil injection, liver biomechanics were evaluated in control mice at 0, 2, and 4 weeks after the initial injection. Representative stress-strain curves at each time point are presented in Supplementary Figure S2A. No significant variations in the Young’s modulus were observed across the three time points (Supplementary Figure S2B).

The increase in the liver’s Young’s modulus is strongly linked to the histological structural changes associated with fibrosis. Our analysis revealed a significant positive correlation between Young’s modulus and the percentage of collagen area (r = 0.7232, p < 0.0001) (Figure 2D). Furthermore, a semi-quantitative assessment of the collagen area fraction derived from SHG images demonstrated a positive correlation with Young’s modulus (r = 0.7080, p < 0.0001) (Figure 2E).

### 3.3 Correlation of activated hepatic stellate cells and molecular expression with liver stiffness

**FIGURE 3:**
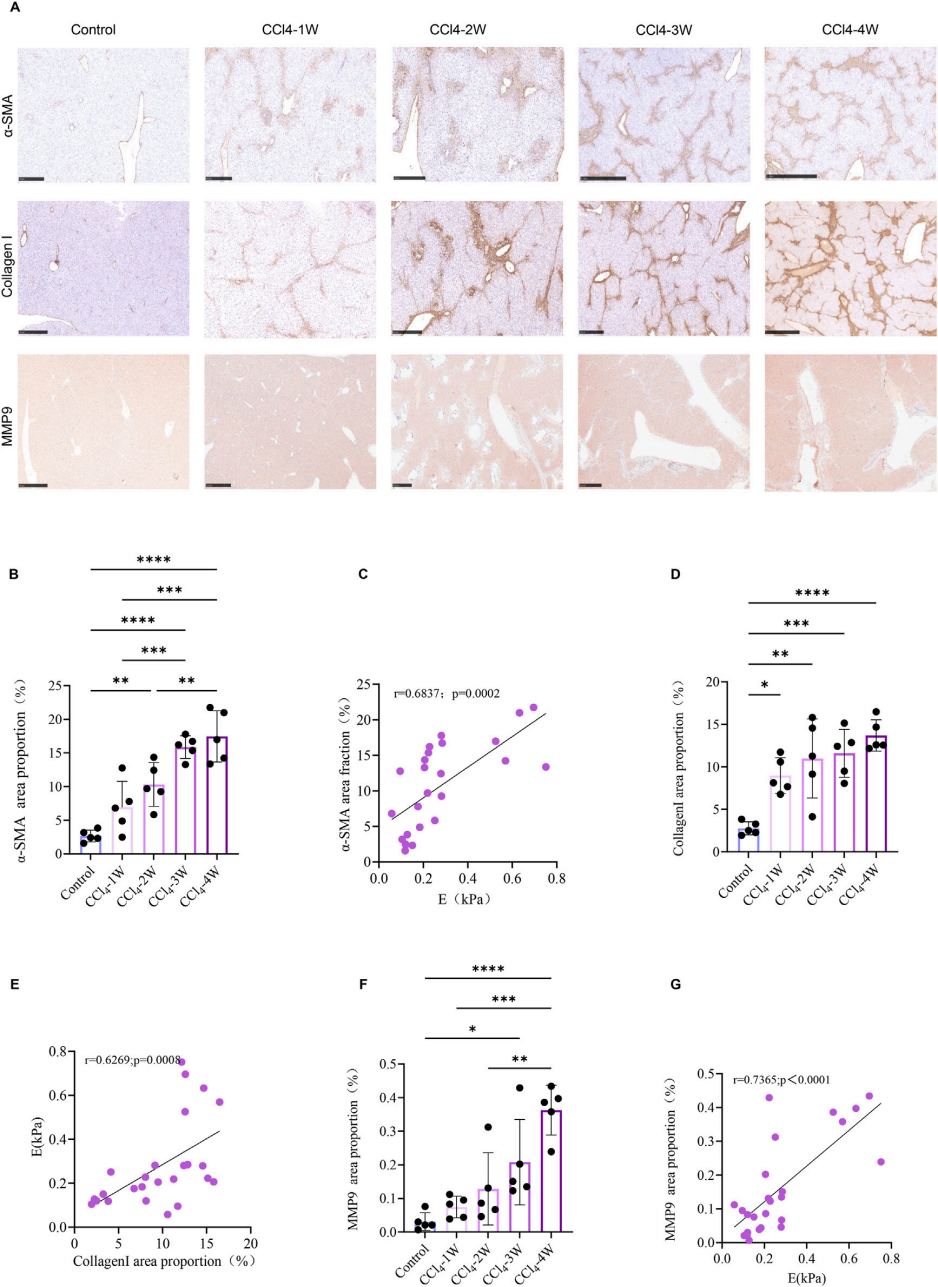
Immunohistochemical staining and statistical results at different stages of the study. **(A)** Representative images of α-SMA, Collagen I, and MMP-9 immunohistochemical staining at indicated time points. **(B)** Quantification of α-SMA-positive area. **(C)** Relationship between Young’s modulus and α-SMA-positive area proportion. **(D)** Quantification of Collagen I-positive area. **(E)** Relationship between Young’s modulus and Collagen I-positive area proportion. **(F)** Quantification of MMP-9-positive area. **(G)** Relationship between Young’s modulus and MMP-9-positive area proportion. Values are expressed as mean ± SD (n = 5 per group). Intergroup comparisons were performed by one-way ANOVA with Tukey’s *post hoc* correction. For correlation analysis, Pearson’s correlation coefficient was used for normally distributed data, and Spearman’s rank correlation coefficient was used for non-normally distributed data. Significance levels: ^∗^p < 0.05; ^∗∗^p < 0.01; ^∗∗∗^p < 0.001; ^∗∗∗∗^p < 0.0001. α-SMA, α-smooth muscle actin; Collagen I, Type I Collagen; MMP-9, Matrix Metallopeptidase-9.

In the control mice, α-SMA immunostaining was confined to the vascular smooth muscle, with negligible positive signals detected in the liver parenchyma No statistically significant variation in α-SMA expression was found between the control groups (Supplementary Figures S3A, B). In contrast, in CCl_4_-treated mice, the α-SMA-positive area was significantly increased (Figure 3A). Positive staining was predominantly observed within the fibrous septa and perisinusoidal spaces. Semi-quantitative analysis indicated a gradual increase in the α-SMA-positive area during CCl_4_ treatment, with statistically significant differences between groups. (Control vs. CCl_4_-2W: p = 0.0045; Control vs. CCl_4_-3W: p < 0.0001; Control vs. CCl_4_-4W: p < 0.0001; CCl_4_-1W vs. CCl_4_-3W: p = 0.001; CCl_4_-1W vs. CCl_4_-4W: p = 0.0001; CCl_4_-2W vs. CCl_4_-4W: p = 0.0082) (Figure 3B). Notably, the α-SMA-positive area fraction showed a positive correlation with Young’s modulus, as revealed by semi-quantitative analysis (r = 0.6837, p = 0.0002) (Figure 3C).

IHC staining for Collagen I in control mice exhibited positive signals exclusively within the blood vessels and portal tracts (Supplementary Figure S3C). Conversely, in mice treated with CCl_4_, Collagen I expression progressively intensified over time, predominantly accumulating within the hepatic sinusoid spaces and outlining the contours of pseudolobules (Figure 3A). Collagen I immunostaining area was significantly increased in CCl_4_-treated versus control mice, as shown by semi-quantitative analysis (Control vs. CCl_4_-1W: p = 0.0148; Control vs. CCl_4_-2W: p = 0.0011; Control vs. CCl_4_-3W: p = 0.0005; Control vs. CCl_4_-4W: p < 0.0001) (Figure 3D). Furthermore, semi-quantitative analysis of the proportion of the Collagen I area demonstrated a positive correlation with Young’s modulus (r = 0.6269; p = 0.0008) (Figure 3E).

MMP-9 immunostaining exhibited minimal positive signals in the control mice, and no significant differences were detected across the control groups (Supplementary Figure S3D). In CCl_4_-treated mice, a notable increase was observed, primarily localized within the inflammatory regions and stromal cells (Control vs. CCl_4_-3W: p = 0.0237; Control vs. CCl_4_-4W: p < 0.0001; CCl_4_-1W vs. CCl_4_-4W: p = 0.0002; CCl_4_-2W vs. CCl_4_-4W: p = 0.0021) (Figure 3F). A semi-quantitative analysis of MMP-9 protein expression levels revealed a significant positive correlation with Young’s modulus values (r = 0.7365, p < 0.0001) (Figure 3G).

### 3.4 Dynamic coupling between hepatic hemodynamics and liver stiffness

#### 3.4.1 Hepatic vascular hemodynamics and spleen morphological changes

**FIGURE 4:**
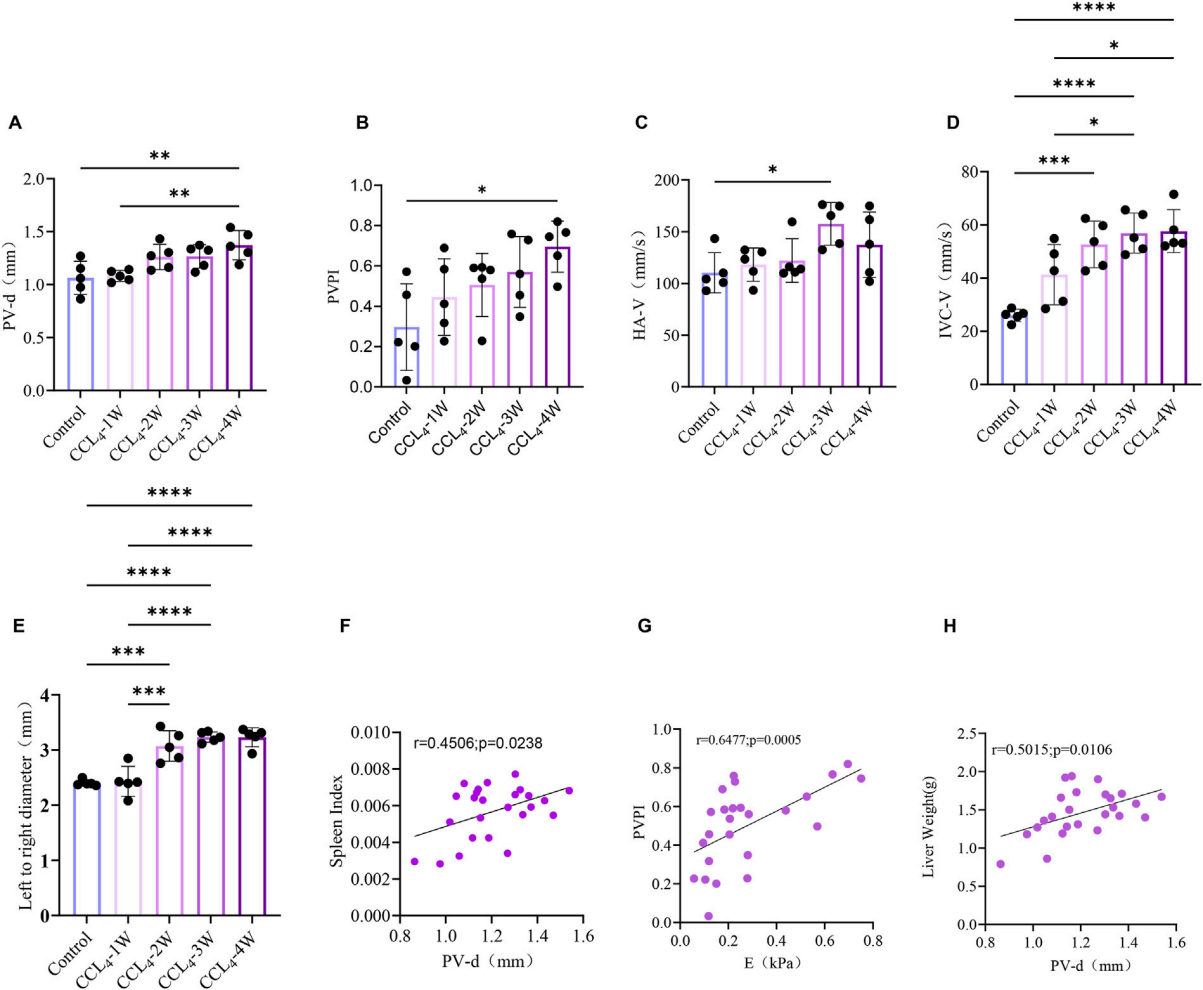
Statistics of abdominal ultrasound-related parameters. **(A)** Portal vein inner diameter statistics. **(B)** Statistical results of the portal venous pulsatility index. **(C)** Statistical results of the hepatic artery mean flow velocity. **(D)** Statistical results of the average flow velocity of the inferior vena cava. **(E)** Statistical results of the spleen left-to-right diameter. **(F)** Correlation analysis of portal vein diameter and splenic index. **(G)** Correlation analysis of the Young’s modulus and portal venous pulsatility index. **(H)** Correlation analysis of liver weight and portal vein diameter. All values are mean ± SD (n = 5 per group). Group differences were determined using one-way ANOVA followed by Tukey’s *post hoc* test. For correlation analysis, Pearson’s correlation coefficient was used for normally distributed data, and Spearman’s rank correlation coefficient was used for non-normally distributed data. ^∗^p < 0.05; ^∗∗^p < 0.01; ^∗∗∗^p < 0.001; ^∗∗∗∗^p < 0.0001. PV-d, Portal vein inner diameter; PVPI, portal venous pulsatility index; HA-V, hepatic artery mean flow velocity; IVC-V, mean inferior vena cava flow velocity.

In the portal hypertension model in mice, both the portal venous and hepatic arterial systems demonstrated dynamic alterations (Supplementary Figure S4A). Portal vein inner diameter showed a progressive rise over the course of the study (Figure 4A), while the mean portal venous flow velocity exhibited an overall decline, notwithstanding a time-dependent increase observed in week 3 (Supplementary Figure S4B). Concurrently, the Doppler derived portal venous pulsatility index displayed a gradual upward trend (Figure 4B). The hepatic arterial flow velocity peaked at week 3 (Figure 4C). Simultaneously, the flow velocity of the inferior vena cava progressively increased during CCl_4_ induction (Figure 4D).

Splenomegaly is a significant morphological marker of portal hypertension. Ultrasound evaluations revealed a progressive increase in the left-to-right diameter of the spleen in CCl_4_-treated mice over time (Figure 4E). Importantly, a moderate positive correlation was observed between the portal vein diameter and splenic index (r = 0.4506, p = 0.0238, q = 0.02539) (Figure 4F). The degree of congestive splenomegaly may correlate directly with the severity of portal hypertension. This finding is directly associated with hemodynamic changes, including portal vein dilation and decreased flow velocity, which correlate with an increased splenic index.

#### 3.4.2 Changes in portal hemodynamics and worsening liver stiffness

To examine the association between altered mechanical properties of liver tissue and hemodynamics, we assessed the correlation between mouse liver Young’s modulus and ultrasound-derived hemodynamic measures. Spearman’s rank correlation showed that Young’s modulus was positively correlated with the portal venous pulsatility index (r = 0.6477, p = 0.0005) (Figure 4G).

To further explore the relationship between hemodynamic changes and organ morphology, we conducted a correlation analysis of the data from weeks 0–4. Pearson’s correlation analysis revealed a moderate positive association of portal vein inner diameter with the liver weight (r = 0.5015, p = 0.0106) (Figure 4H).

### 3.5 Biochemical criterion

#### 3.5.1 Hepatocyte injury and liver stiffness

**FIGURE 5:**
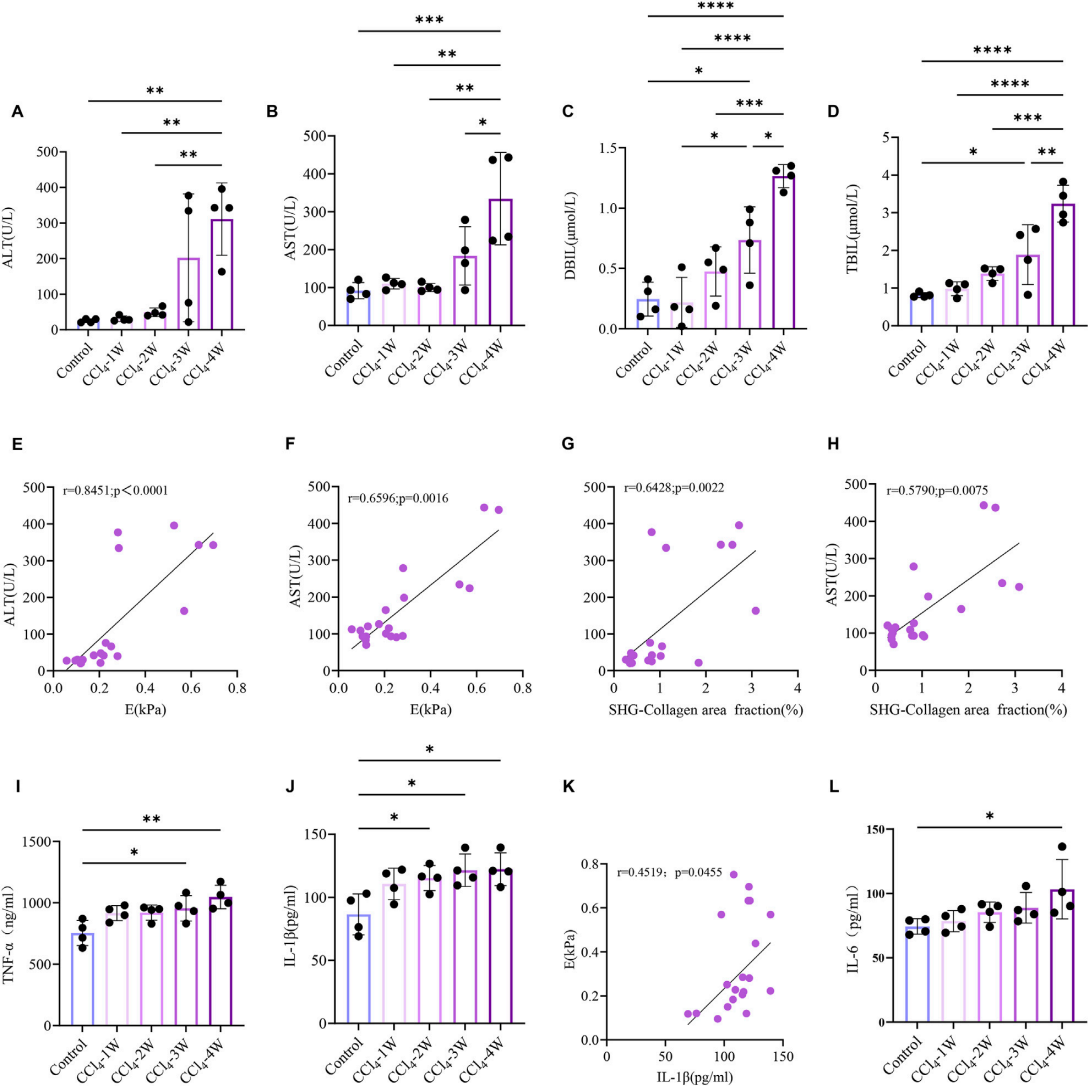
Liver cell damage, systemic inflammatory response, and increased tissue stiffness are all related to each other. **(A)** Serum ALT statistical analysis. **(B)** Serum AST statistical analysis. **(C)** Statistical results for serum DBIL concentrations. **(D)** Serum TBIL concentrations. **(E)** Correlation analysis between E and ALT levels. **(F)** AST levels versus E. **(G)** ALT levels versus collagen area percentage. **(H)** AST levels versus collagen area percentage. **(I)** Statistical analysis of Serum TNF-α levels. **(J)** Statistical analysis of Serum IL-1β levels. **(K)** Correlation analysis between IL-1β and E. **(L)** Statistical analysis of Serum IL-6 levels. Data represent mean ± SD (n = 4 per group). One-way ANOVA and Tukey’s *post hoc* test were employed for multiple group comparisons. For correlation analysis, variables with a normal distribution were evaluated using Pearson’s correlation coefficient, while non-normally distributed variables were analyzed using Spearman’s rank correlation coefficient. ^∗^p < 0.05; ^∗∗^p < 0.01; ^∗∗∗^p < 0.001; ^∗∗∗∗^p < 0.0001. ALT, alanine aminotransferase; AST, aspartate aminotransferase; TBIL, total bilirubin; DBIL, direct Bilirubin; IL-1β, interleukin-1β; IL-6, interleukin-6; TNF-α, tumor necrosis factor-α

To quantify hepatocyte damage, we measured serum ALT, AST, DBIL, and TBIL. With the prolonged CCl_4_ treatment, ALT and AST gradually increased, while TBIL and DBIL only significantly increased in the later stages of the experiment (Figures 5A–D). No statistically significant differences were detected among the control groups (Supplementary Figures S5A–D). Both ALT and AST levels demonstrated a positive correlation with the liver’s Young’s modulus (ALT: r = 0.8451, p < 0.001; AST: r = 0.6596, p = 0.0016) and with collagen area fraction (ALT: r = 0.6428, p = 0.0022; AST: r = 0.5790, p = 0.0075) (Figures 5E–H).

Analysis of serum TNF-α concentrations revealed that TNF-α levels in the CCl_4_-treated group began to increase in the first week of induction and remained elevated during weeks 3-4, showing statistically significant differences compared to the control group (Figure 5I). No statistical differences were observed among the control groups (Supplementary Figure S4E).

Serum IL-1β levels demonstrated a time-dependent trend in the CCl_4_-treated group (Figure 5.J). Concentrations at weeks 2, 3, and 4 were significantly higher than those in the control group (p < 0.05). All control groups showed comparable values, with no significant differences detected (Supplementary Figure S5F). Pearson’s correlation analysis between serum IL-1β concentrations and liver Young’s modulus in mice demonstrated a moderate positive correlation (r = 0.4519; p = 0.0455, q = 0.0455) (Figure 5K).

At the conclusion of the model induction period, IL-6 levels were also significantly higher than those in the control group, with a statistically significant difference (Figure 5L). No statistical differences were observed among the control groups (Supplementary Figure S5G).

#### 3.5.2 Serum markers of portal hypertension and their correlation with liver stiffness

**FIGURE 6:**
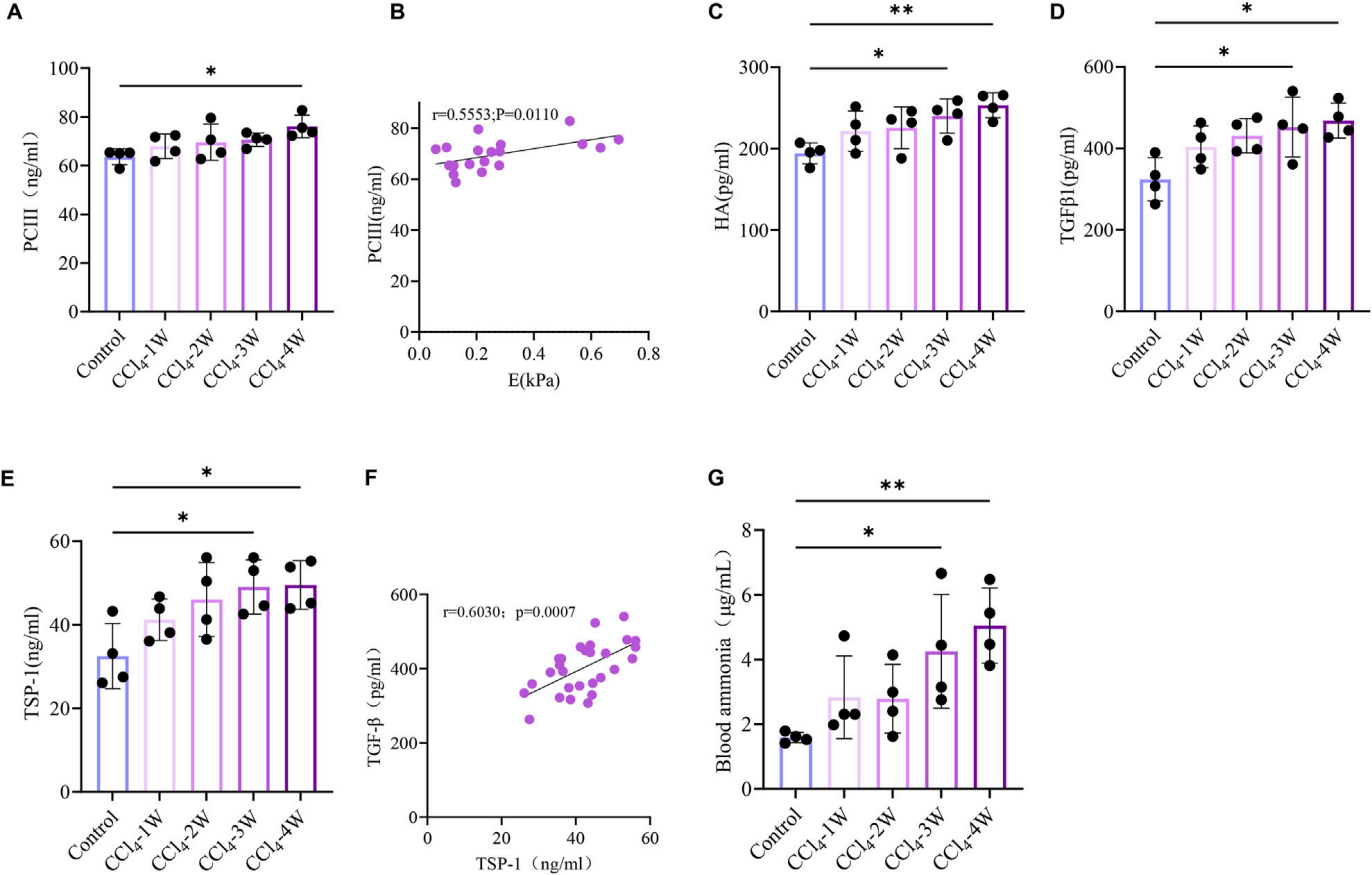
Serum Activity Indicators and Statistics of Portal Hypertension. **(A)** Serum PC-III levels. **(B)** Correlation between E and serum PC-III levels. **(C)** Serum HA levels. **(D)** Serum TGF-β1 levels. **(E)** Serum TSP-1 levels. **(F)** Correlation between TSP-1 and TGF-β1 levels. **(G)** Serum blood ammonia levels. Data are presented as mean ± SD (n = 4 per group). Statistical significance was assessed using one-way analysis of variance (ANOVA), followed by Tukey’s *post hoc* test to evaluate differences between groups. For correlation analysis, variables with a normal distribution were evaluated using Pearson’s correlation coefficient, while non-normally distributed variables were analyzed using Spearman’s rank correlation coefficient. ^∗^p < 0.05; ^∗∗^p < 0.01. PC-III, pro-type III collagen; HA, hyaluronic acid; TGF-β1, transforming growth factor-β; TSP-1, thrombospodin-1.

The CCl_4_-4W group showed significantly higher serum levels of type III procollagen (PC-III) than the control group (Figure 6A), with no statistical differences observed among the control groups (Supplementary Figure S5H). Correlation analysis with Young’s modulus demonstrated a moderately strong positive correlation (r = 0.5553, p = 0.0110, q = 0.0126) (Figure 6B). HA levels were elevated at weeks 3 and 4 in CCl_4_-treated mice compared to control mice (Control vs. CCl_4_-3W; p = 0.0457; Control vs. CCl_4_-4W: p = 0.0078) (Figure 6C), with no statistical differences observed among the control groups (Supplementary Figure S5I).

TGF-β1 levels progressively increased during CCl_4_ induction, with statistically significant differences observed at weeks 3 and 4 compared to the control group (p < 0.05) (Figure 6D), while no significant intergroup differences were found across the control groups (Supplementary Figure S5J). Serum TSP-1 levels were significantly increased in CCl_4_-treated mice (Figure 6.E), with no statistical differences among the control groups (Supplementary Figure S5K). Notably, serum TSP-1 levels correlated positively with total TGF-β1 levels (r = 0.6030, p = 0.0007) (Figure 6.F). This association is biologically plausible, as TSP-1 is a well-established activator of latent TGF-β1 through its ability to bind the latency-associated peptide (LAP) and induce conformational changes that release active TGF-β1 (Hayashi and Sakai, 2012). The TSP-1–TGF-β1 axis has been implicated in fibrogenesis and extracellular matrix remodeling in various tissues, including the liver. Serum ammonia concentrations were significantly elevated at weeks 3 and 4 compared to those in the control group (Figure 6G), indicating impaired hepatic detoxification, with no statistical differences among control groups (Supplementary Figure S5L).

## 4 Discussion

Portal hypertension drives cirrhosis morbidity (Ramkissoon et al., 2024; Long et al., 2025), yet systematic biomechanical studies are few. Our multimodal longitudinal data show that progressive liver stiffness correlates with rising portal resistance, collagen deposition, and hepatic stellate cell activation. One major limitation is the lack of direct measurement of portal vein pressure; we used ultrasound to measure the portal venous pulsatility index (PVPI) as an alternative. Thus, findings are correlational, not causal. It should be noted that olive-oil control groups at weeks 1 and 3 were not included; therefore, a time-dependent contribution of the vehicle at the intermediate time points where CCl_4_ effects peaked (weeks 1 and 3) cannot be fully excluded. However, control groups at weeks 0, 2, and 4 showed stable values across all parameters, suggesting that long-term vehicle effects are unlikely. The observed fibrosis-stiffness-resistance sequence is consistent with a mechanobiological hypothesis that excessive matrix deposition may compress sinusoids and activate stellate cells, perpetuating intrahepatic resistance. This hypothesis awaits interventional testing. Our main novelty is the integrated multiscale, time-resolved dataset. Although repeated preconditioning is widely recommended for soft tissues to reduce cycle-to-cycle variability and obtain a reproducible steady-state mechanical response, the objective of the present study was different. Rather than characterizing the steady-state constitutive behavior, we aimed to quantify the initial tensile response of freshly excised murine liver under a standardized loading history. Representative stress–strain curves from repeated loading (Supplementary Figure S2D) showed a modest stress-softening effect after the first loading cycle. However, repeated loading inevitably increases the cumulative loading history imposed on this highly hydrated and compliant tissue, which may promote fluid redistribution and microstructural reorganization during *ex vivo* testing. To minimize such cumulative alterations and preserve the initial *ex vivo* mechanical state as much as possible, a single standardized preconditioning cycle was applied to all specimens before formal testing.

To place our measurements in the context of previous studies, we compared our Young’s modulus values with published data obtained using different experimental protocols. Barnes et al. (2007) investigated fresh *ex vivo* livers from adult C57BL/6 mice (8–10 weeks) using indentation combined with finite-element reconstruction and reported an elastic modulus of approximately 0.6 kPa for normal liver tissue. In contrast, Estermann et al. (2021). Measured fresh murine liver using uniaxial tensile testing and reported Young’s modulus values in the 10–20 kPa range, while also demonstrating that tissue preservation (e.g., Thiel fixation) substantially altered the measured mechanical properties. Reiter et al. (2021) evaluated adult mouse liver *in vivo* using multifrequency magnetic resonance elastography (MRE), reporting frequency-dependent shear stiffness values that increased with fibrosis progression. Collectively, these studies show that the reported stiffness values obtained by different mechanical testing techniques span more than one order of magnitude. Such variability is expected because liver tissue exhibits pronounced viscoelasticity and its measured stiffness is highly sensitive to experimental methodology, including loading mode (tension, compression, indentation, or MRE), strain rate, pre-conditioning protocol, specimen geometry (especially thickness), and tissue status (fresh, preserved, or *in vivo*). Therefore, the differences between our measurements and previously reported values are most likely attributable to these methodological factors rather than reflecting inconsistencies in the mechanical properties of murine liver itself.

Progression in cirrhosis-induced portal hypertension is intricately linked with liver biomechanics. Felli et al. (2023) and Chang et al. (2023) posited that the viscoelastic multiscale mechanical indices and their distribution characteristics exhibit significant variation in response to liver fibrosis and mesenchymal stem cell (MSC) therapy, suggesting that such mechanical parameters may help assess how the condition evolves and what lies ahead for the patient. Building on this premise, we conducted a quantitative assessment of the impact of cirrhotic portal hypertension on the macroscopic mechanical properties of hepatic tissue through uniaxial tensile testing of *ex vivo* liver specimens. Analysis of the stress-strain curves indicated a progressive increase in the Young’s modulus of the liver tissue corresponding to the severity of cirrhosis and portal hypertension. Previous research has successfully achieved three-dimensional reconstruction and analysis of collagen structures during liver cirrhosis using SHG (Liu et al., 2017). Consistently, our combined analysis utilizing Masson’s trichrome staining and SHG imaging demonstrated that the collagen fiber area fraction was positively associated with the Young’s modulus of the liver. Notably, PCIII levels were positively correlated with Young’s modulus. These findings suggest that increased collagen deposition during liver fibrosis is the primary structural basis for alterations in macroscopic liver mechanics. This finding may have clinical translational implications, confirming that the core material basis of liver stiffness, as measured by non-invasive liver elastography, is the abnormal deposition of the intrahepatic collagen fiber network, thereby validating the biological efficacy of this technology. According to De Smet et al. (2021) among patients with acute liver failure, the degree of HSC activation and markers of hepatocyte injury both show significant associations with liver stiffness. Concurrently, in the field of hepatology, some studies have suggested that liver stiffness may serve as a surrogate marker for the degree of HSC activation and the severity of liver injury (Lee et al., 2014). In the present study, immunohistochemical analysis of α-SMA, Collagen I, and MMP-9 revealed that their expression levels correlated positively with liver stiffness. These results suggest that a stiff microenvironment is associated with HSC activation, which may in turn contribute to ECM crosslinking and deposition. This process could create a feed-forward cycle that amplifies liver stiffness, although this interpretation remains correlational.

The parallel elevation of MMP-9 with stiffness (Figures 3F,G) is noteworthy. Although MMP-9 is canonically matrix-degrading, its context-dependent role in fibrosis is recognized. Its prominent immunostaining in inflammatory infiltrates (Figure 3A) suggests a major contribution from macrophages/neutrophils rather than HSCs. This inflammatory MMP-9 might disrupt sinusoidal architecture and basement membrane remodeling, potentially facilitating myofibroblast migration and activation, rather than counterbalancing the increased ECM deposition. The net effect could thus favor ECM accumulation and stiffening.

Cirrhosis-induced portal hypertension progression is associated with significant hemodynamic alterations (de Franchis et al., 2022; Andrés-Rozas et al., 2025). Ultrasound evaluation of liver-related vascular parameters revealed an overall decrease in the mean portal vein velocity, with a time-dependent increase observed at week 3 of treatment. Hepatic artery velocity reached its peak at week 3, whereas inferior vena cava velocity exhibited a general upward trend with intergroup variability. These findings indicate the presence of splanchnic hyperdynamic circulation and compensatory enhancement of the hepatic arterial buffer response. Mesineni et al. (2025) recently characterized cirrhosis as a multisystem disease, wherein the primary driving mechanism is splanchnic vasodilation and a hyperdynamic circulatory state induced by portal hypertension. This conceptual framework provides a basis for understanding the redistribution of blood flow within the splanchnic circulation. Our observations are supported by progressive portal vein dilation and splenomegaly during disease progression, as well as a positive correlation between portal vein diameter and spleen size. Young’s modulus also correlated positively with the portal venous pulsatility index, suggesting a link between liver stiffness and portal hypertension severity. This finding is consistent with the hypothesis that increased tissue stiffness may elevate intrahepatic vascular resistance. Overall, these results indicate that altered hepatic biomechanics may contribute to portal hypertension pathogenesis, pending further validation.

Cappelli et al. (2025) reported that in patients with chronic liver disease, Young’s modulus is independently associated with AST levels, an effect thought to reflect necroinflammatory activity. In our mouse model, we similarly observed that elevations in ALT and AST paralleled the increase in Young’s modulus over time. These parallel observations across species are consistent with, but do not prove, a link between hepatocellular injury and time-dependent stiffness changes. Accordingly, we examined serum biomarkers from a biochemical standpoint. Serum TGF-β1 pointed to persistent fibroblast activation. During disease progression, initial elevations in ALT and AST were accompanied by a steady increase in Young’s modulus, indicating worsening liver injury. Later, TBIL and DBIL levels rose markedly, suggesting that this mechanically driven process had moved toward intrahepatic cholestasis.

Serum ALT and AST levels showed a linear association with liver Young’s modulus and collagen area, suggesting a possible link among hepatocyte injury, extracellular matrix deposition, and increased tissue stiffness. Consistent with existing literature, elevated ALT and AST levels, which serve as sensitive indicators of hepatocyte injury, are indicative of liver inflammation (Hendy et al., 2017). Persistent inflammation has been identified as a key driver of hepatocyte activation, promoting abnormal ECM deposition (Widowati et al., 2026). This excessive ECM deposition is biomechanically manifested as increased liver stiffness (Lv et al., 2026). Notably, alterations in the mechanical microenvironment can activate hepatic stellate cells through integrin-mediated pathways, establishing a positive feedback loop among inflammation, fibrosis, and mechanical signal transduction (Wong et al., 2022). Supporting this, we observed a positive association between serum IL-1β levels and liver Young’s modulus, suggesting that mechanical signals may amplify local inflammatory responses via Kupffer cell activation and inflammatory cell recruitment (Li et al., 2021). Concurrently, the elevated HA levels are consistent with possible sinusoidal capillarization, while the increased blood ammonia may reflect functional portal-systemic shunting and reduced hepatic detoxification capacity.

Importantly, all correlations reported in this study should be interpreted as associations rather than causal relationships. The temporal sequence is consistent with, but does not prove, a mechanistic link. These biomarker changes together associate microcirculatory structural remodeling with systemic metabolic disturbances, offering further insight into the pathophysiology of portal hypertension in cirrhosis.

Although previous studies have documented variations in liver viscoelasticity across different species and environments (Estermann et al., 2021), to the best of our knowledge, this study is among the first to systematically integrate liver stiffness with hemodynamic parameters, histopathological features, and biochemical indices in an animal model of PH. We developed an integrated mechanical-hemodynamic model of cirrhotic portal hypertension. HSC activation is associated with pathological ECM deposition and remodeling, which may contribute to increased liver stiffness. This rigid microenvironment may contribute to sinusoidal and microvascular compression, potentially increasing intrahepatic resistance. Such a sequence is consistent with the hypothesis that stiffness could be one of the factors that facilitate portal hypertension progression, but this interpretation remains correlational and awaits causal validation through interventional studies.

In summary, this study provides a comprehensive, time-resolved correlational dataset linking liver stiffness to portal hemodynamic deterioration in a mouse cirrhosis model. While causality requires invasive pressure monitoring and interventional experiments, our findings support the mechanobiological hypothesis and offer a reference for future studies targeting tissue stiffness as a therapeutic avenue.

## Data Availability

The original contributions presented in the study are included in the article/Supplementary Material, further inquiries can be directed to the corresponding authors.
