# Supplementary material for: Multimodal assessment of liver stiffness and portal hemodynamics in a cirrhotic mouse model
Source: Front Bioeng Biotechnol. 2026 Aug 3;14:1888436. doi: 10.3389/fbioe.2026.1888436 (PMC13478080; doi:10.3389/fbioe.2026.1888436)
Supplement: Supplementary file 1 [file Supplementaryfile1.pdf]

Multimodal Assessment of Liver Stiffness and Portal Hemodynamics in a Cirrhotic Mouse Model

Jingtian Zhang[1,2], Yujie Huo[1,2], Jingxuan Bian[1,2], Li Cha[1,2], Qiyue Yang[1], Chunjuan Zhao[1], Jinyu Li[1], Dongliang Zhao[3*], Duiping Feng[1*], Long Gao[1*]

[1] Department of Oncological and Vascular Intervention, First Hospital of Shanxi Medical University, Taiyuan 030001, Shanxi Province, China

[2] Department of Medical Imaging, Shanxi Medical University, Taiyuan 030001, Shanxi Province, China

[3] Institute of Biomedical Engineering, Shenzhen Bay Laboratory, Shenzhen, Guangdong, China

Table of contents

Supplementary methods 1

Supplementary figures 4

Supplementary references 10

1 Supplementary methods

1.1 Immunohistochemical staining

Target	Company Catalog No.	Dilution	Host	Retrieval buffer (pH) & method	Retrieval time
α-SMA	Abcamab124964	1:10000	Rabbit	Citrate, pH 6.0, high-pressure	2min
Collagen I	Abcamab270993	1:200	Rabbit	EDTA, pH 9.0, high-pressure	1min30s
MMP-9	bosterPB9669	1:500	Rabbit	EDTA, pH 9.0, high-pressure	1min30s

Goat anti-rabbit IgG-HRP (Abcam, ab205718; 1:2000; 37°C, 45 min). DAB substrate kit (Maxim, DAB-4033; used according to manufacturer's instructions)

Paraffin-embedded sections were deparaffinized in eco-dewaxing solution (three changes, 10 min each) and rehydrated through graded ethanol (anhydrous, 95%, 75%; 5 min each), followed by three washes with TBS (3 min each). Antigen retrieval was performed using a pressure cooker: for α-SMA, sections were heated in citrate buffer (pH 6.0) for 2 min; for Collagen I and MMP-9, sections were heated in EDTA buffer (pH 9.0) for 1 min 30 s. After cooling to room temperature (25–30°C, 1 h), sections were washed with TBS (3 × 5 min). Endogenous peroxidase activity was blocked with 3% H_2O_2 for 30 min at room temperature. Sections were then circled with a hydrophobic pen, washed with TBST, and blocked with 10% normal goat serum in TBST for 30 min at room temperature. Primary antibodies diluted in 10% normal goat serum were applied (α-SMA, 1:10,000; Collagen I, 1:200; MMP-9, 1:500) and incubated overnight at 4°C. The next day, sections were brought to room temperature for 15 min, washed with TBST (3 × 3 min), and incubated with HRP-conjugated goat anti-rabbit secondary antibody (1:2000) for 45 min at 37°C. After washing, freshly prepared DAB substrate was applied, and color development was monitored under a microscope. The reaction was stopped by rinsing with tap water. Sections were counterstained with Harris hematoxylin for 1 min, differentiated in 1% acid-alcohol for 1–2 s, blued, dehydrated in absolute ethanol, air-dried or dried at 37°C, and mounted with eco-mounting medium. Negative controls were performed by replacing the primary antibody with normal rabbit serum or TBST, and positive controls were run in parallel for each antibody using tissue sections known to express the respective antigens: mouse aorta for α-SMA, mouse skin for Collagen I, and MMP-9 with mouse kidney.

1.2 Image quantification

Quantitative image analysis was performed using ImageJ (version 1.54p, for Windows 64-bit, National Institutes of Health, USA). All quantifications were conducted by two independent investigators blinded to the experimental groups, and the average values were used for statistical analysis. For the red pulp area fraction in the spleen, images were converted to HSB color space (HSB Stack). The Hue channel was used to isolate red pulp regions, and the threshold was automatically determined using the Huang algorithm. The area fraction was calculated as (positive area / total tissue area) × 100%.(Schindelin et al., 2012) For immunohistochemistry, RGB images were split into separate color channels (split channels). For each antibody, the same color channel was analyzed across all sections. Positive staining was segmented using either the Huang or Li automatic threshold method, and the method was kept consistent for all images of the same antibody.(Ruifrok and Johnston, 2001) The area fraction was calculated as described above. For second harmonic generation (SHG) imaging of collagen and Masson's trichrome staining, RGB stacks were processed without prior color space conver-

sion. The Otsu method was applied for SHG images, and the Li method was used for Masson's trichrome images to define positive areas.(Hong et al., 2020) All threshold parameters were fixed for each staining type across all samples.

1.3 Serum analysis

All ELISA kits were used according to the manufacturers' instructions. Biochemical parameters were measured using a Mindray BS-240VET fully automated veterinary biochemical analyzer with the corresponding Mindray reagents.

Parameter	Manufacturer	Catalog No.
PC-III	MEIMIAN (Guangzhou, China)	MM-44669M2
HA	JINGMEI (Jiangsu, China)	JM-061638O1
IL-1β	JINGMEI (Jiangsu, China)	JM-02323M2
IL-6	JINGMEI (Jiangsu, China)	JM-02446M2
TNF-α	JINGMEI (Jiangsu, China)	JM-02415M2
TGF-β1	MEIMIAN (Guangzhou, China)	MM-0135M2
TSP-1	MEIMIAN (Guangzhou, China)	MM-0327M2
Ammonia	JINGMEI (Jiangsu, China)	ADS-W-N021
TBIL	Mindray (Shenzhen, China)	105-020584-00
ALT	Mindray (Shenzhen, China)	105-020579-00
DBIL	Mindray (Shenzhen, China)	105-00021A-00
AST	Mindray (Shenzhen, China)	105-020580-00

2 Supplementary Table

Variable	Shapiro-Wilk W	Shapiro-Wilk p
Liver Weight	0.9629	0.4753
PV-d	0.9874	0.9841
TGF-β	0.9835	0.9236
TSP-1	0.9649	0.4517

Table S1. Normality assessment of continuous variables used in correlation analyses

3 Supplementary figures

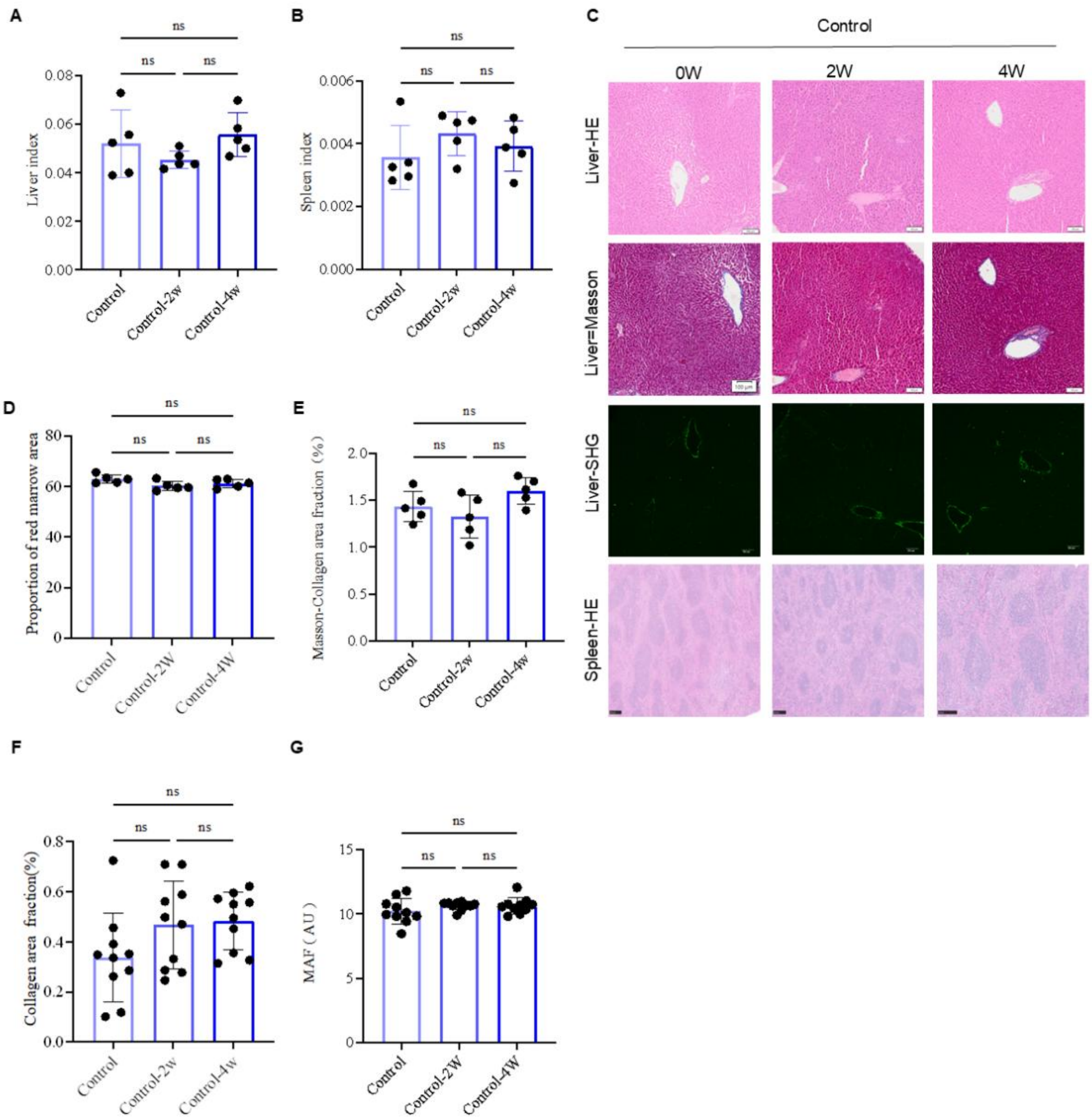

Fig.S1. Changes in liver and spleen indices and staining-related parameters in control groups. (A) Liver index (liver weight / body weight × 100%). (B) Spleen index (spleen weight / body weight × 100%). (C) Representative histopathological images of the liver (HE, Masson's trichrome, SHG) and spleen (HE) at different time points. (D) Quantitative analysis of the proportion of red pulp area in the spleen based on H&E staining. (E) Quantification of the collagen-positive area in the liver using Masson's staining. (F) Quantification of the collagen-positive area in the liver using SHG imaging. (G) Quantification of the mean autofluorescence intensity in the liver based on SHG imaging. Olive oil-treated mice were evaluated at weeks 0, 2, and 4. No significant differences were observed among the three time points across all parameters. Data are presented as mean ± SD (n = 5 per group). Statistical significance was determined using one-way analysis of variance (ANOVA) followed by Tukey's post hoc test. ns, $p > 0.05$.

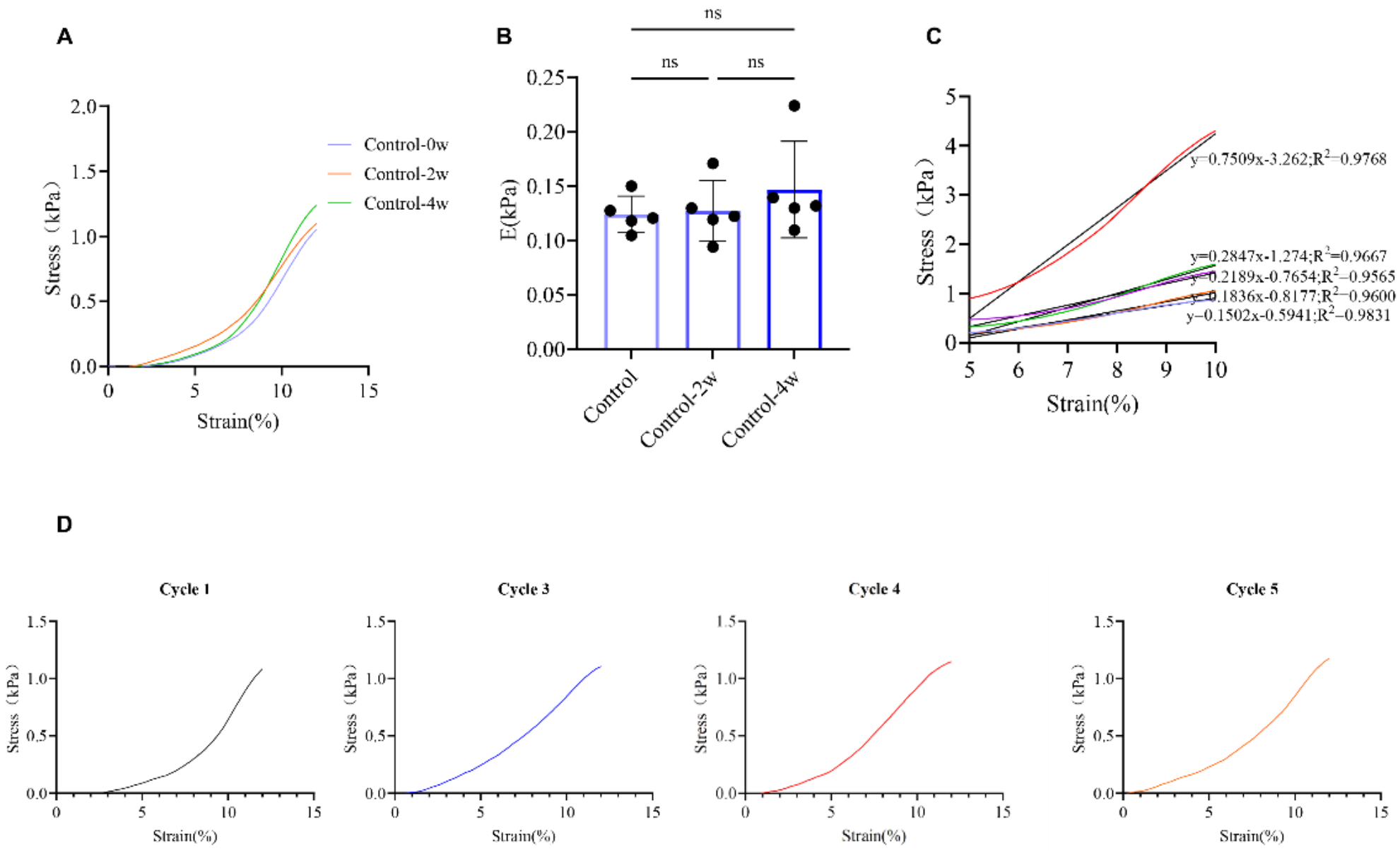

Fig.S2. Liver biomechanics remained stable in olive oil–treated control mice over 4 weeks and linear validation in the 5–10% strain range. (A) Representative stress-strain curves of liver tissues from control mice at weeks 0, 2, and 4. (B) Quantitative analysis of Young's modulus showed no significant differences among the three time points. (C) Representative stress–strain curves for each experimental group are shown. The linear part used to calculate Young's modulus (5–10% strain) is highlighted in color. The linear fit equation and R^2 value for each representative curve are also indicated (all $R^2 > 0.95$). (D) Stress-strain curves for preconditioning cycles 1 vs 3-5 times. Data are presented as mean ± SD (n = 5 per group). Statistical significance was determined by one-way analysis of variance (ANOVA) followed by Tukey's post hoc test. ns, $p > 0.05$.

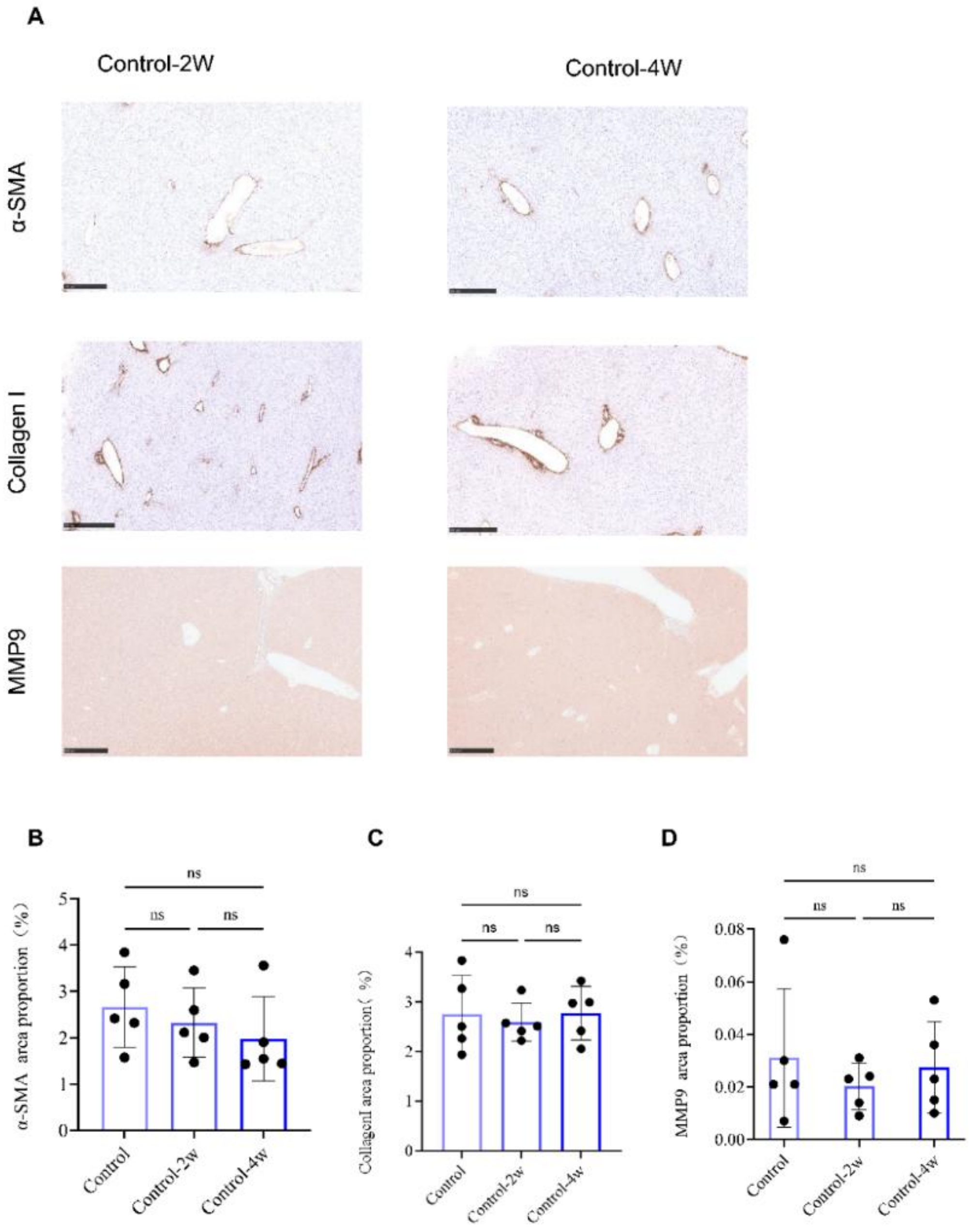

Fig.S3. Grouped histological images and quantification of positive expression levels in the control groups are shown. No statistically significant differences were observed in the expression of HSC activation markers (α-SMA and collagen I) or MMP9 in the olive oil–treated control group at 0, 2, and 4 weeks. Data are presented as mean ± SD ($n = 5$ per group). Statistical analysis was performed using one-way analysis of variance (ANOVA) followed by Tukey's post hoc test. ns, $p > 0.05$.

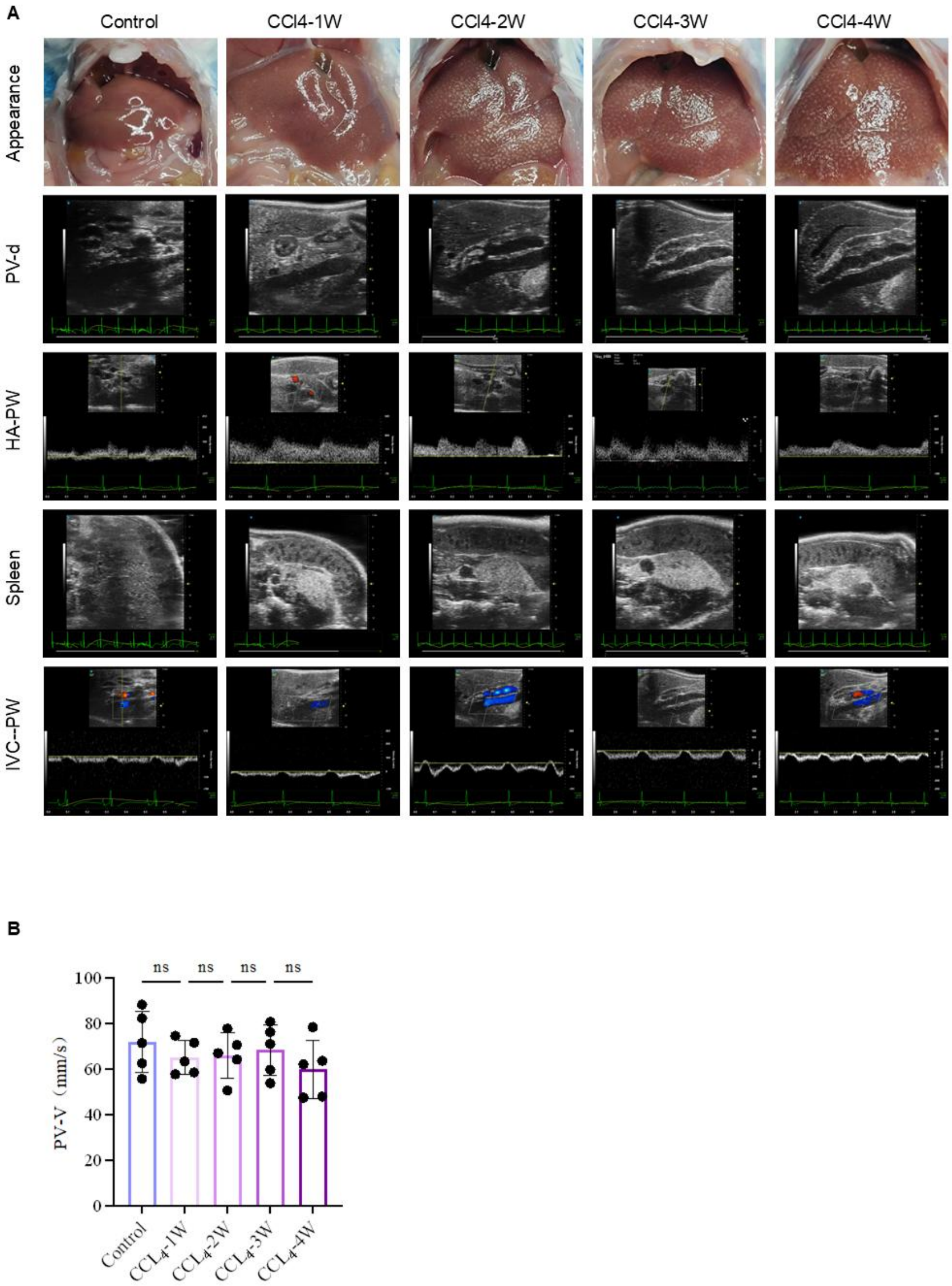

Fig.S4. Mouse abdominal ultrasound image. (A) Gross morphology and representative ultrasound images of the portal vein, hepatic artery, spleen, and inferior vena cava in mice treated with CCl_4 for 1–4 weeks, compared with week 0 controls. (B) Quantitative analysis of portal vein mean velocity. Data are presented as mean ± SD (n = 5 per group). Statistical significance was determined by one-way analysis of variance (ANOVA) followed by Tukey's post hoc test for multiple comparisons. ns, $p > 0.05$.

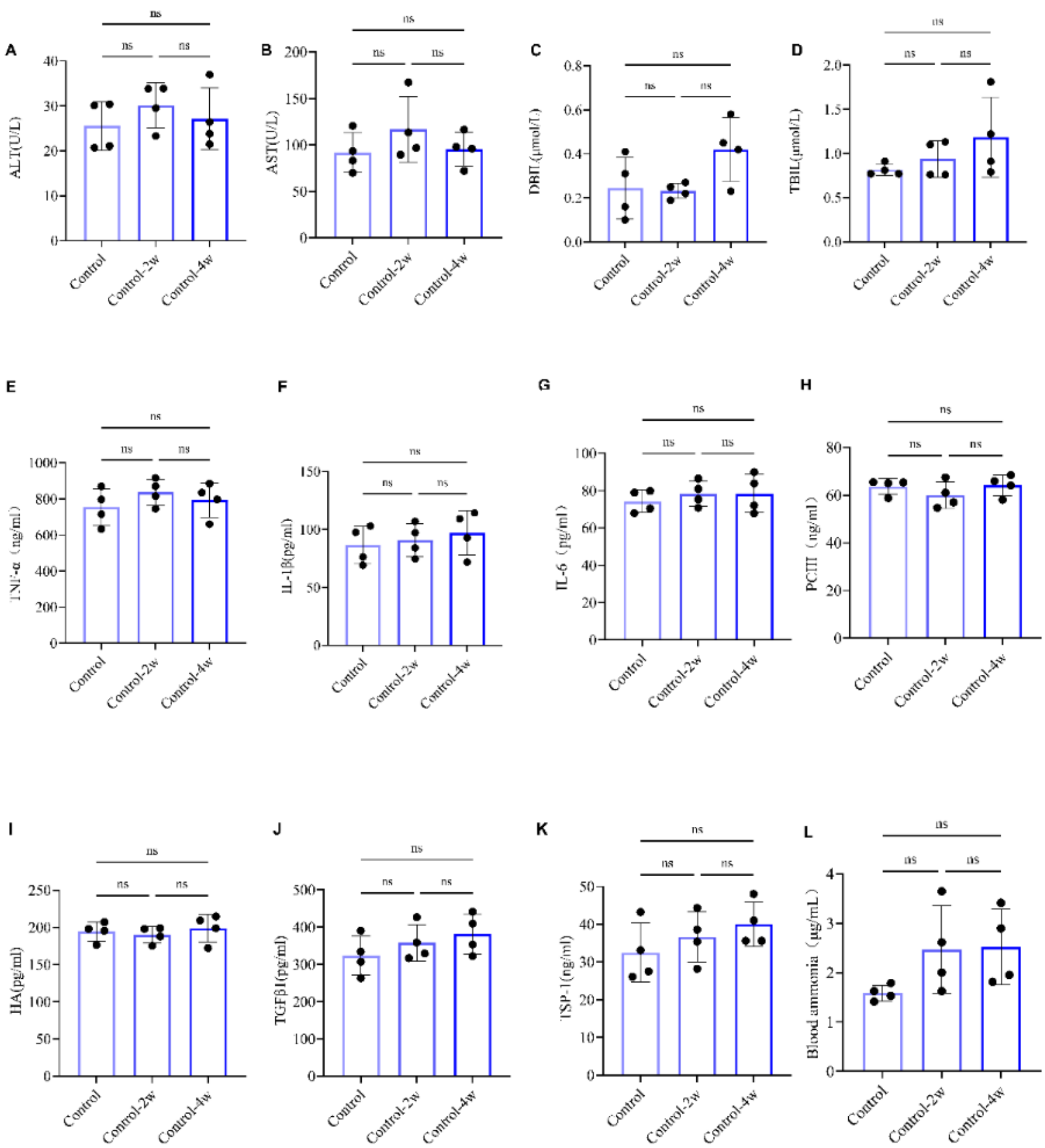

Fig.S5. Serum indicator statistical analysis. Mice were administered olive oil at 0, 2, and 4 weeks, and the corresponding serum indicators were quantitatively analyzed. No statistically significant differences were observed between the groups. Data are presented as mean ± SD (n = 4 per group). Statistical significance was determined by one-way analysis of variance (ANOVA) followed by Tukey's post hoc test for multiple comparisons. ns, $p > 0.05$.

Supplementary references

HONG, J. H., KIM, D. H., RHYU, I. J., KYE, Y. C. & AHN, H. H. 2020. A simple morphometric analysis method for dermal microstructure using color thresholding and moments. *Skin Res Technol,* 26**,** 132-136.

RUIFROK, A. C. & JOHNSTON, D. A. 2001. Quantification of histochemical staining by color deconvolution. *Anal Quant Cytol Histol,* 23**,** 291-9.

SCHINDELIN, J., ARGANDA-CARRERAS, I., FRISE, E., KAYNIG, V., LONGAIR, M., PIETZSCH, T., et al. 2012. Fiji: an open-source platform for biological-image analysis. *Nat Methods,* 9**,** 676-82.
